# Oncocytes in Mucoepidermoid Carcinoma of the Palate: Diagnostic Challenges

**DOI:** 10.1155/2017/5741821

**Published:** 2017-12-28

**Authors:** Rammohan Kumar, Srikant Natarajan, K. S. Sneha, Nunna Sai Chitra, Karen Boaz, Nidhi Manaktala

**Affiliations:** ^1^Oral and Maxillofacial Surgeon, TCR Multispeciality Hospital, Krishnagiri, Tamilnadu, India; ^2^Department of Oral Pathology and Microbiology, Manipal College of Dental Sciences, Manipal Academy of Higher Education, Mangalore, India

## Abstract

The origin of a salivary gland tumour is attributed to cells at various levels of differentiation which present histologically as diverse tissues and cellular patterns. Mitochondria-rich, eosinophilic oncocytes are cells commonly encountered in salivary gland neoplasms. We report a case of mucoepidermoid carcinoma (MEC) in the palate of a 43-year-old female that exhibited a prominent oncocytic component. While the parotid and submandibular glands have been reported as predominant sites for oncocytic MEC (OMEC), the palate and minor salivary glands are rare sites for occurrence. Also, most of the reported cases of OMEC have been histologically of low-grade mucoepidermoid carcinoma with large cystic spaces and good prognosis. In this article, we discuss the differential diagnosis and diagnostic workup of an MEC presenting with oncocytes.

## 1. Introduction

Oncocytes are large epithelial cells that contain bright eosinophilic, granular cytoplasm. The term “oncocyte” is derived from the Greek word “*onkousthai*” which means swollen or enlarged. The oncocyte was first described by Schaffer in 1897 who observed the presence of eosinophilic granular cells in ductal and acinar elements of salivary glands of the tongue, pharynx, and esophagus. He believed that oncocytes were the result of a degenerative phenomenon in salivary gland parenchymal cells. Zimmermann observed the presence of oncocytes in the sublingual gland and referred to these cells as “pyknocytes” alluding to their condensed nuclear chromatin or pyknotic nuclei [1]. Hamperl used the term “*onkocyte*” to describe large granular cells seen not only in the salivary gland tissue but also in tissues of the kidney, thyroid, parathyroid, pituitary, and adrenal glands. He suggested that individual cells or aggregates of cells could either undergo permanent modification into oncocytes as a metaplastic process or proliferate as a hyperplastic or neoplastic process [1]. Oncocytes have since been observed in the liver, pancreas, fallopian tubes, testes, stomach, and bronchi. Ultrastructural studies of tumours showing oncocytes have demonstrated increased numbers of mitochondria within the cytoplasm of the oncocyte. Histochemical studies performed to compare the concentrations of oxidative enzymes of the oncocyte to those of normal salivary gland acinar and ductal cells have shown similarities between an oncocyte and the intercalated duct reserve cell. However, some ultrastructural studies suggest that the salivary gland oncocyte may be an adaptive or compensatory hyperplastic cell occurring secondary to an undetermined somatic mutation rather than a purely degenerative process [1].

The World Health Organization (2005) classified oncocytic lesions into three distinct categories of oncocytosis, oncocytoma, and oncocytic carcinoma. Other lesions described to show oncocytes include both benign and malignant tumours like oncocytic/oncocytoid variants of cystadenoma, myoepithelioma, acinic cell carcinoma, salivary duct carcinoma, and metastatic renal cell carcinoma [2]. Oncocytic metaplasia is a feature of Warthin's tumour and may also be a component of malignant salivary gland tumours like mucoepidermoid carcinoma, acinic cell carcinoma, pleomorphic adenoma, and oncocytic myoepithelioma [3].

In the current article, we describe the case of a low-grade mucoepidermoid carcinoma of the palate with numerous oncocytes.

## 2. Case Report

**Figure 1:**
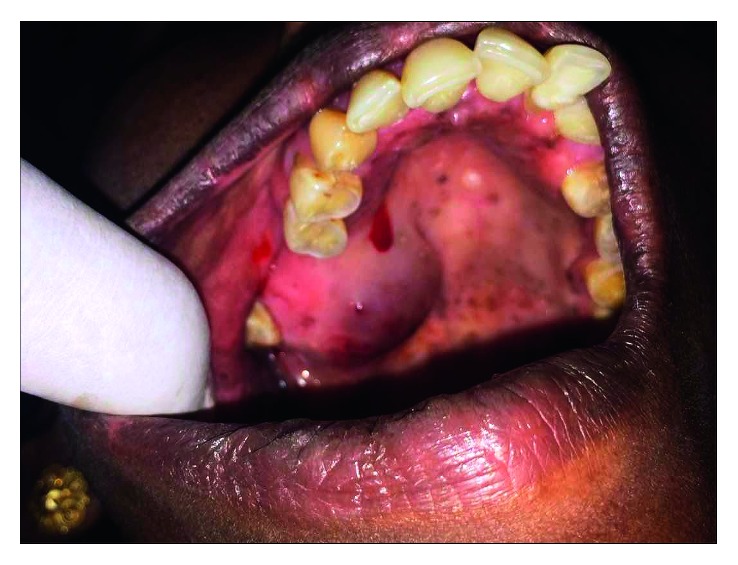
Soft fluctuant swelling in the right posterior part of the palate. Spontaneous bleeding was noted from the lesion.

A 43-year-old female patient reported to a private clinic with a soft fluctuant swelling extending anteroposteriorly from the second premolar to the third molar in the right side of the palate. She had noticed a small swelling in the same site 3 years back, which was suspected to be a periodontal abscess, and the adjacent tooth was extracted. However, the swelling did not subside and gradually grew to attain the size of 4 × 3 cm. Adjacent teeth were intact and firm (Figure 1).

An incisional biopsy exhibited fibrous connective tissue stroma with predominance of mucicarmine-positive mucous cells with pale foamy cytoplasm intermixed with intermediate, epidermoid, clear, and oncocytic cells. Small gland-like structures were also seen along with few engorged blood vessels and areas of hemorrhage. Based on the histological features, a diagnosis of low-grade mucoepidermoid carcinoma was made. Following this, right hemimaxillectomy was performed, and the specimen was sent for histopathological examination.

### 2.1. Gross Appearance

The excisional specimen consisted of a resected palate including teeth 15, 16, and 17 with associated soft tissues measuring approximately “4.4 × 3.5 × 3.0” cm. An ulcer which was measuring approximately “0.8 × 0.6” cm was noticed on the hard palate in relation to the medial aspect of 16.

### 2.2. Microscopic Appearance

**Figure 2:**
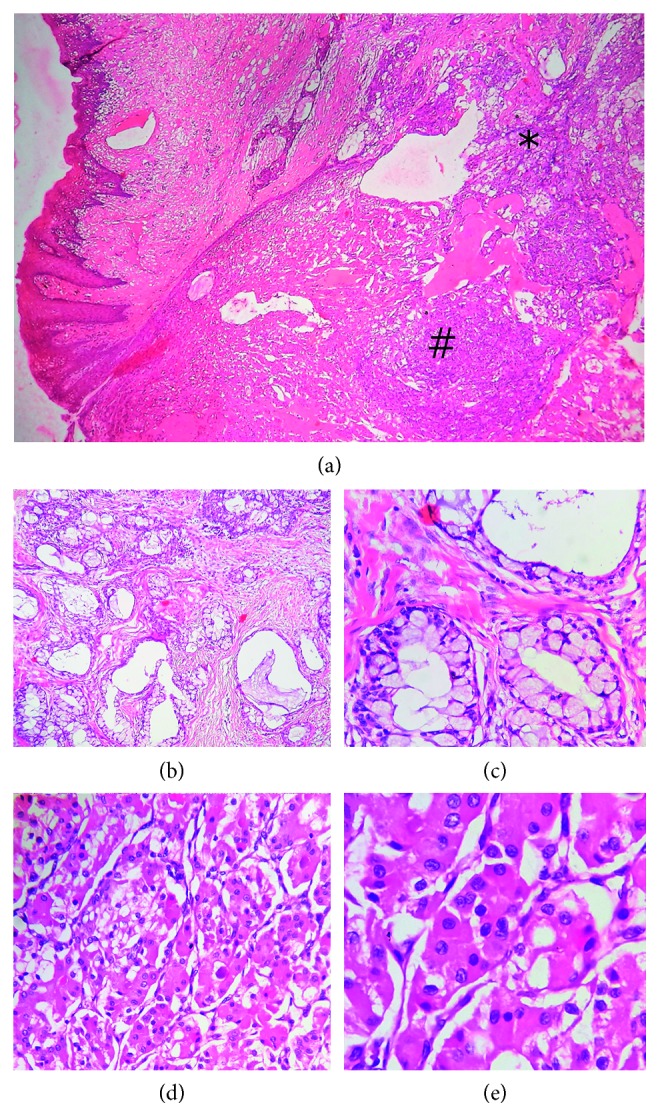
(a) Low-power view showing neoplastic glandular epithelial cells (∗) separated from the overlying mucosal parakeratinized stratified squamous epithelium. Oncocytic cells (#) intermixed with the cellular elements of mucous, epidermoid, and intermediate cells (magnification 4x). (b) Tumour mass showing multiple large cystic spaces (magnification 10x). (c) Cystic spaces lined by mucous cells (magnification 40x). (d) Oncocytic metaplasia evidenced by the presence of oncocytes (magnification 10x). (e) Cells with granular eosinophilic cytoplasm arranged in an organoid/alveolar pattern and supported by thin fibrous connective tissue septa (magnification 40x).

Microscopic examination in low power showed the presence of neoplastic glandular epithelial cells that were separated from the overlying mucosal parakeratinized stratified squamous epithelium. Oncocytic cells were also seen to be intermixed with the cellular elements of mucous, epidermoid, and intermediate cells (Figure 2). On higher magnification, the lesional tissue (tumour mass) was seen to exhibit large cystic spaces that were lined by mucous, epidermoid, intermediate, spindle-shaped, and few clear cells (Figures 2 and 2). Oncocytic metaplasia was evidenced as extensive areas of polyhedral cells with granular eosinophilic cytoplasm arranged in an organoid/alveolar pattern and supported by thin fibrous connective tissue septa (Figures 2 and 2). The connective tissue stroma surrounding the tumour mass exhibited a moderate amount of chronic inflammatory cell infiltrates, salivary gland acini, focal areas of hyalinization, engorged blood vessels, and adipocytes.

The presence of large cystic spaces lined by the classic population of three types of cells (i.e., mucous, epidermoid, and intermediate cells) accompanied by a predominance of oncocytic cells led us to the diagnosis of a low-grade mucoepidermoid carcinoma with oncocytic metaplasia.

## 3. Discussion

Mucoepidermoid carcinoma (MEC) is the most common malignant salivary gland neoplasm of the major and minor salivary glands. An intraosseous variant of the MEC has also been described. MECs are characterized by the presence of three types of cells: mucous, intermediate, and epidermoid cells in varying proportions. Minor proportion of the histology may also be constituted by clear, columnar, or oncocytic cells.

Less frequently encountered variants of the MEC include oncocytic MEC, sebaceous MEC, psammomatous MEC, spindle cell MEC, goblet cell aggressive MEC, and sclerosing MEC. Although the variants do not appear to affect the prognosis of the tumour, the predominance of a less-frequent histological cell type does pose a challenge to diagnose MEC, as it may obscure the classical histopathological features. One such variant is the oncocytic mucoepidermoid carcinoma (OMEC) showing high proportion of oncocytes. Oncocytic change is not only a neoplastic event but also a component of the normal aging process. Thus, an MEC containing oncocytes may put the pathologist in a dilemma to categorize it as an OMEC or MEC with oncocytic metaplasia. The percentage of oncocytes in the lesion to discriminate these two has not been defined in the literature owing to the rarity of this variant and the relatively fewer number of studies documented. However, Brannon and Willard defined a tumour with classical histopathological pattern of the MEC with mucous, epidermoid, and intermediate cells with ≥50% showing oncocytic changes as OMEC [4]. Weinreb et al. studied all the MEC tumours containing oncocytes and included only those cases with >50% oncocytic elements as OMEC [3]. Jahan-Parwar et al. described 3 cases of the MEC (2 low-grade and 1 high-grade) containing >75% and >60% oncocytes, respectively [5].

MEC is the most common malignant salivary gland tumour which histologically shows the presence of mucous, intermediate, and epidermoid cells. Low-grade lesions are characterized by the presence of cystic spaces with abundance of mucous cells. High-grade lesions, on the other hand, show solid areas of intermediate and epidermoid cells with presence of necrosis and neural involvement. Low-grade MECs with presence of oncocytes are not difficult to distinguish from other oncocytoid tumours as areas of typical MEC are invariably present within the tumour. When oncocytic cells predominate with few epidermoid and intermediate cells, the diagnosis becomes difficult and other oncocytic lesions should be considered in the differential diagnosis. The lesions showing the presence of oncocytes can be broadly grouped as

1. oncocytic lesions which include oncocytosis, oncocytoma, and oncocytic carcinoma;
2. benign lesions like pleomorphic adenoma, Warthin's tumour, and oncocytic myoepithelioma which shows oncocytic metaplasia;
3. malignant lesions exhibiting oncocytes which include mucoepidermoid carcinoma and acinic cell carcinoma;
4. metastatic lesions with oncocytes which include hepatocellular carcinoma, thyroid carcinoma, and renal cell carcinoma [3].

As with all pathologies, adequate sampling of the excised specimen is needed to eliminate errors in diagnosis that may occur due to restricted/selective sampling.

The distinguishing features of these oncocytic lesions are summarized in Table 1.

Oncocytic MEC has been reported to occur most often in the parotid gland [6], followed by the submandibular gland [7], and only in 3 cases in the minor salivary glands. Of these cases, individuals from the 2nd to the 8th decade were affected with a slight male predominance. All cases of OMEC were of low grade showing large cystic spaces histologically with a good prognosis [4]. The pathogenesis of the oncocytic change in salivary glands has been attributed to degenerative cell changes, metaplastic transformation, or an adaptive/compensatory mechanism secondary to an undetermined somatic mutation [1].

A lesion showing predominance of oncocytes obscuring the classical histological features of the MEC may be difficult to diagnose based on routine histopathology. The primary aim would be to identify the lesion as malignant. Studies have shown that p63 is a reliable marker to help in the distinction of OMEC from other tumours. The rationale for using p63 in the diagnosis of OMEC is its expression in the epidermoid component of the conventional MEC as opposed to the peripheral staining pattern in oncocytoma and oncocytic carcinomas [3]. Recently, studies have shown that MECs result from a recurrent t(11,19)(q21;p23) translocation resulting in an MECT1-MAML2 fusion [8]. This fusion gene can be used to identify and arrive at a diagnosis of MEC in cases where the tumour is composed predominantly of oncocytes [9].

## 4. Conclusion

A pathologist must remain cognizant of the rare diagnosis of oncocytic mucoepidermoid carcinoma of the palate which has a good clinical prognosis. Although oncocytic changes are predominantly age-related or seen in benign lesions, similar change may be occasionally encountered in oncocytic carcinoma or oncocytic mucoepidermoid carcinoma. It may be worthwhile to additionally assess the expression of p63 in diagnosing these malignant oncocytic lesions especially in the absence of a reliable criterion to histopathologically differentiate metaplastic and neoplastic oncocytic lesions.

## Figures and Tables

**Table 1:** Difference between OMEC and other oncocytic lesions.

S. no.	Lesion	Microscopic appearance	
(1)	Oncocytic mucoepidermoid carcinoma	Predominance of oncocytes showing granular eosinophilic cytoplasm along with large cystic structures lined by mucous cells, epidermoid cells, and intermediate cells	
—	*Lesion*	*Microscopic appearance*	*Differentiating feature from OMEC*
(2)	Oncocytosis	Unencapsulated foci of oncocytic cells appearing in multiple separate nodules. The lesion contains residual (nononcocytic) salivary gland parenchyma	Absence of epidermoid, mucous, and intermediate cells either focally or predominantly
(3)	Oncocytoma	Encapsulated tumour exhibiting an organoid/alveolar growth pattern separated by thin fibrous connective tissue septa	Encapsulation. Absence of mucous, epidermoid, and intermediate cells
(4)	Oncocytic carcinoma	Unencapsulated, single, or multinodular tumour. Oncocytic cells exhibit pleomorphism. Infiltration into the salivary gland parenchyma in the form of trabeculae, sheets, and nests	Absence of mucous, epidermoid, and intermediate cells and presence of atypical features
(5)	Pleomorphic adenoma	Encapsulated, presence of chondromyxoid areas, characteristic melting pattern seen	Encapsulation. Chondromyxoid areas, presence of ducts
(6)	Warthin's tumour	Papillary cystic lesion with bilayered oncocytic epithelium overlying lymphoid stroma	Absence of mucous cells, not infiltrative
(7)	Acinic cell carcinoma	Forms a solitary mass or multiple nodules and invades in broad fronts. Predominantly cellular with less fibrous stroma. Tumour cells arranged in organoid sheets which are traversed by ramifying blood vessels. Prominent growth pattern: microcyst pattern	Absence of/minimal fibrous stroma. Absence of mucous cells, epidermoid, and intermediate cells
